# Administrative data deficiencies plague understanding of the magnitude of rape-related crimes in Indian women and girls

**DOI:** 10.1186/s12889-022-13182-0

**Published:** 2022-04-19

**Authors:** Rakhi Dandona, Aradhita Gupta, Sibin George, Somy Kishan, G. Anil Kumar

**Affiliations:** 1 Public Health Foundation of India, Gurugram, India; 2 Institute for Health Metrics and Evaluation, University of Washington, Seattle, USA

## Abstract

**Background:**

This paper investigates trends in rape-related crimes against women and girls reported in the Indian administrative data from 2001 to 2018 to assess the burden of crime, describe sub-national variations, and highlight data gaps to address sexual violence effectively in India.

**Methods:**

Data on five rape-related crimes were extracted from the annual reports of National Crimes Record Bureau (NCRB), and included assault with the intent to outrage modesty of woman, rape, insult to the modesty of women, attempt to commit rape, and murder with rape/gang-rape. Rates for all categories combined, and for each crime were estimated for women and girls for India and its states. Trends for type of offender for rape, mean number of people arrested, and legal status of the cases was also assessed.

**Results:**

The rate of all rape-related crime increased from 11.6 in 2001 to 19.8 in 2018 per 100,000 women and girls. Most of the 70.7% increase in rate between 2001 and 2018 was post 2012 following a gang-rape and murder case in India’s capital. The largest proportion of crimes was recorded as assault with the intent to outrage modesty of the woman, followed by rape. The cited offender in rape cases was for the majority a close known person (44·3%) or other known person (43·1%). By the end of 2018, only 9·6% of the cases had completed trials, with acquittals in 73% cases.

**Conclusions:**

The wide variations in the yearly crime rates at state-level highlighted significant issues in data quality including under-reporting, non-comparability, possible bias in data reporting in NCRB, definition of rape-related crime in India, and access in reporting of crimes. Addressing barriers to reporting, improving quality and scope of administrative data recorded on sexual violence is urgently needed for India to meet SDG targets of eliminating all forms of violence against women and girls.

**Supplementary Information:**

The online version contains supplementary material available at 10.1186/s12889-022-13182-0.

## Background

Violence against women, both by intimate partner (IPV) and non-partner, is a public health concern [1]. The Sustainable Development Goal (SDG) target five is to eliminate all forms of violence against women and girls, and the two indicators of progress towards this are the rates of IPV and non-partner violence [2]. Acknowledging the issue’s large scale and extensive consequences, many countries have passed laws criminalising violence, and are increasingly providing legal, health, and social services to the abused women.

The most recent significant discontent on sexual violence against women in India was brought by the *Nirbhaya* case, which was gang rape with fatal assault of a 23-year old woman in December 2012 in New Delhi, the capital of India [3–5]. This case catalysed widespread public protests in major cities throughout the country against the state and central governments for failing to provide adequate security for women [6]. The judicial committee set up to investigate the case indicated that failures on the part of the government and police were the root cause behind crimes against women in India [7]. Following this, the Indian government passed several new sexual assault laws, including a mandatory minimum sentence of 20 years for gang rape and six new fast-track courts created solely for rape prosecutions under the Criminal Law (Amendment) Ordinance in 2013 [8]. Since then, four other gang rape cases have incited similar protests in India, two of which were in the year 2020 [9–12]. Much of the literature on sexual violence in India addresses its societal context, [3, 13–17] with little systematic understanding available on the burden of sexual violence to guide a public health approach aimed at reduction to reach the SDG target [2].

The official statistics on all types of accidents and crimes, including sexual violence, for each state are reported by the National Crime Record Bureau (NCRB) (https://ncrb.gov.in/en/crime-india). India is a large country with 1.3 billion diverse population across 29 states and seven union territories; with population of many states larger than some countries [18]. These states vary in their socio-demographic profile, which contributes to varied burden and pattern of various diseases and injuries as documented elsewhere [19, 20]. Furthermore, health is a state subject in India, and policy- and program-making is done at the level to improve the wellbeing of the people [21]. In this background we analysed the NCRB data on rape-related crimes against women and girls in India from 2001 to 2018 to assess the trend over time in the reported rape-related crimes and to describe the scale of state (sub-national) variations, and to identify the data issues that need attention to facilitate effective public health interventions to reduce sexual violence in India at the state-level.

## Methods

The administrative data on rape in India are consolidated yearly by the NCRB under the “Crime in India report”. It’s primary data source is police records from India’s states and Union Territories, submitted in the NCRB prescribed proforma through a software application developed by NCRB [22]. We utilised the 2001 to 2018 reports available in the public domain for this analysis.

Our original aim of this analysis was to understand sexual violence against women only and not girls, and hence we downloaded the tables detailing data on rape-related crimes against women which are available in NCRB under five categories - assault on women with intent to outrage her modesty, rape, insult to the modesty of women, attempt to commit rape, and murder with rape/gang rape. The definitions of these crime categories as per the Indian Penal Code (IPC) are shown in Supplementary Table 1 [23]. However, detailed review of data on age of the cases reported under these categories showed that age categories were available only for rape cases for all the years, and age was not available for any other categories other than for assault on women with intent to outrage her modesty for 2017-18 (Supplementary Table 2). Also, the age categories under rape were not consistent over time and showed overlap of age. In this background, we then considered all cases irrespective of age for this analysis, and present analysis for women and girls. Furthermore, India also enacted a specific Act (*Protection of Children from Sexual Offences Act; POCSO*) for victims under 18 years of age, [24] and data under this act was available for years 2017 and 2018. However, this age group was also covered under the rape category for 2017 and 2018, and it was not possible to exclude duplication of cases as individual level data were not available. Therefore, we did not include cases registered under POCSO Act in this analysis.

The number of cases registered under each of the five rape-related crime categories were extracted for the years 2001 to 2018 for each state and India. The NCRB follows the “Principle Offence Rule” to classify each case irrespective of the number of offences under which it is legally registered [22], which means that each case in the report is unique and is filed under the most serious crime. We extracted the numbers of persons arrested under each category, available from 2001 to 2015 for the states, and until 2018 for overall India. The type of offender which was reported only for rape cases was also extracted; and the reporting categories in NCRB have changed over time. For this analysis, we classified the type of offender into four categories for ease of analysis and interpretation – family, close known person, known person, and unknown person (Table 1). Finally, we extracted data on the number of legal cases filed for the rape-related crimes and their current status in the judicial system. The legal data were available cumulatively for only India and because it was not possible to extrapolate these for each year from the tables, we use only the most recent year for these data – 2018.

**Table 1:** Re-categorisation of type of offender for rape-related crimes available in the National Crimes Records Bureau (NCRB)

Type of offender classification used for analyses	NCRB categories
Family	• Parents / close family members • Grandfather / father / brother / son, etc^a^ • Close family members^a^ • Family members^b^ • Live in partner / husband (separated /ex)^**c**^
Close known person	• Relative • Neighbours • Employer / co-worker^a^ • Known persons on promise to marry the victim^c^ • Family friends / neighbours / employers or other known persons^b^ • Friends / online friends or live in partners on pretext of marriage / separated husband^b^
Other known person	• Other known persons^d^
Unknown person	• Offenders not identified by victim^c^ • Offenders not known to victim^e^ • Cases offender unknown or could not be identified^b^

^a^Sub-category added in NCRB in 2014

^b^Sub-category added in NCRB in 2017

^c^Sub-category added in NCRB in 2015

^d^Not available in NCRB for 2017 and 2018

^e^Sub-category added in NCRB in 2016

The primary aim of this analysis was to examine the trends in rape-related crime rates against women from 2001 to 2018 across three administrative splits: nationally, by groups of state, and individual state. The states were assigned into three groups based on the socio-demographic index (SDI) computed by the Global Burden of Disease (GBD) study which is based on lag distributed income, average years of education for population > 15 years of age, and total fertility rate [25]. We categorised the states using the SDI index to ensure the comparability of the findings of this analysis with the findings reported under the India State Burden Initiative, which is addressing the distribution of major diseases/conditions and risk factors at the state-level in India [26–28].

For the purpose of this analysis, we used the GBD study 2019 state-wise estimated annual population for women and girls as the denominator to estimate the crime rate instead of the population denominator used by the NCRB to maintain consistency of comparison with the other published GBD analysis at the state-level for India [20, 26–28]. We report these crime rates for 100,000 women and girls with 95% confidence intervals. It is important to note that the estimated crime rates as reported in the NCRB reports and those estimated using GBD study population were similar (data not shown). We analysed the type of offenders for rape cases from 2001 to 2018 for India and for states from 2001 to 2015 as per the data availability. Furthermore, we analysed the changes over the years in the mean number of people arrested for rape-related crime, which was estimated by dividing the number of persons arrested by the total number of cases reported for each year. Lastly, we report on the status of legal cases filed for these crimes for India in 2018. As this analysis used aggregated data available in public domain, no ethics approval was necessary. The statistical analysis was done using MS excel 2016 and the maps were created using QGIS [29].

## Results

### All rape-related crimes

**Fig. 1:**
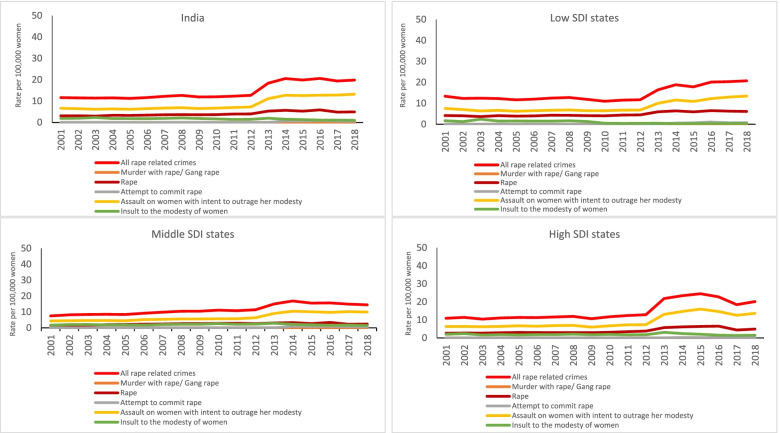
Yearly trends in rape-related crime rate per 100,000 women and girls reported in NCRB, 2001–2018. SDI denotes Socio-demographic Index

A total of 1,597,466 rape-related crime cases were reported in India from 2001 to 2018. The NCRB reported 59,945 cases in 2001 which increased to 133,836 in 2018 in India, corresponding to all rape-related crime rate of 11.6 (95% CI 11.5–11.7) in 2001 to 19.8 (95% CI 19.7–19.9) per 100,000 women and girls in 2018. This rate documented a significant increase of 70.7% (95% CI 69·5–71·9) between 2001 and 2018 (Table 2 and Fig. 1). The rate of all rape-related crimes in the Indian states ranged from 1.8 (95% CI 1.7–1.9) in Bihar to 49.4 (95% CI 48.5–50.3) in Odisha per 100,000 women and girls in 2018 (Table 3), with this rate being slightly but significantly higher in the low SDI (13.4; 95% CI 13.2–13.5) as compared with the middle- and high- SDI states as shown in Table 2.

**Table 2:** Rate of rape-related crimes reported in NCRB per 100,000 women and girls in India and by the Socio-demographic Index (SDI) state groups. CI denotes confidence interval

	2001 (95% CI)	2005 (95% CI)	2010 (95% CI)	2015 (95% CI)	2018 (95% CI)	% change 2001–2018
**India**						
All rape-related crimes	11.6 (11.5–11.7)	11.2 (11.2–11.3)	12.0 (11.9–12.1)	19.8 (19.7–20.0)	19.8 (19.7–19.9)	70.7
Assault on women with intent to outrage her modesty	6.61 (6.54–6.68)	6.15 (6.08–6.21)	6.70 (6.64–6.77)	12.6 (12.5–12.6)	13.2 (13.1–13.3)	99.7
Rape	3.11 (3.07–3.16)	3.30 (3.25–3.35)	3.66 (3.61–3.71)	5.28 (5.23–5.34)	4.94 (4.89–5.00)	58.7
Insult to the modesty of women	1.89 (1.85–1.93)	1.80 (1.76–1.83)	1.64 (1.61–1.68)	1.32 (1.30–1.35)	1.04 (1.01–1.06)	−45.1
Murder with gang rape^a^	Not available	Not available	Not available	Not available	0.04 (0.04–0.05)	
Attempt to commit rape^b^	Not available	Not available	Not available	0.7 (0.7–0.7)	0.6 (0.6–0.6)	
**Low SDI states**						
All rape-related crimes	13.4 (13.2–13.5)	11.6 (11.5–11.8)	11.0 (10.9–11.1)	17.8 (17.7–18.0)	20.7 (20.6–20.9)	55
Assault on women with intent to outrage her modesty	7.5 (7.4–7.6)	6.2 (6.1–6.3)	6.5 (6.4–6.5)	10.9 (10.8–11.0)	13.4 (13.3–13.6)	78.5
Rape	4.15 (4.07–4.23)	3.87 (3.80–3.95)	4.03 (3.96–4.10)	5.9 (5.8–6.0)	6.1 (6.0–6.2)	47.3
Insult to the modesty of women	1.69 (1.64–1.75)	1.57 (1.52–1.62)	0.49 (0.47–0.52)	0.36 (0.34–0.39)	0.38 (0.36–0.40)	−77.6
Murder with gang rape^a^	Not available	Not available	Not available	Not available	0.06 (0.05–0.07)	
Attempt to commit rape^b^	Not available	Not available	Not available	0.68 (0.65–0.71)	0.74 (0.71–0.77)	
**Middle SDI states**						
All rape-related crimes	9.5 (9.3–9.6)	10.5 (10.4–10.7)	13.9 (13.8–14.1)	19.4 (19.2–19.6)	18.0 (17.8–18.2)	90.5
Assault on women with intent to outrage her modesty	5.4 (5.3–5.5)	5.6 (5.5–5.7)	7.1 (7.0–7.2)	12.6 (12.4–12.8)	12.4 (12.3–12.6)	129.0
Rape	1.92 (1.85–1.99)	2.63 (2.55–2.70)	3.51 (3.42–3.60)	3.34 (3.26–3.42)	2.96 (2.89–3.04)	54.1
Insult to the modesty of women	2.12 (2.05–2.19)	2.30 (2.22–2.37)	3.32 (3.23–3.40)	2.40 (2.33–2.47)	1.84 (1.78–1.90)	−13.2
Murder with gang rape^a^	Not available	Not available	Not available	Not available	0.04 (0.02–0.05)	
Attempt to commit rape^b^	Not available	Not available	Not available	1.09 (1.04–1.13)	0.77 (0.73–0.81)	
**High SDI states**						
All rape-related crimes	10.9 (10.7–11.0)	11.3 (11.1–11.5)	11.7 (11.5–11.9)	24.5 (24.2–24.7)	20.1 (19.9–20.3)	85.4
Assault on women with intent to outrage her modesty	6.3 (6.1–6.4)	6.7 (6.5–6.8)	6.7 (6.6–6.8)	16.0 (15.8–16.2)	13.6 (13.5–13.8)	116.9
Rape	2.6 (2.5–2.7)	3.0 (2.9–3.1)	3.1 (3.0–3.2)	6.3 (6.2–6.5)	4.9 (4.8–5.0)	88.3
Insult to the modesty of women	1.98 (1.90–2.05)	1.63 (1.56–1.69)	1.92 (1.85–1.99)	2.00 (1.93–2.07)	1.45 (1.39–1.50)	−26.9
Murder with gang rape^a^	Not available	Not available	Not available	Not available	0.03 (0.01–0.04)	
Attempt to commit rape^b^	Not available	Not available	Not available	0.18 (0.16–0.20)	0.14 (0.12–0.15)	

^a^Data reported for 2017 and 2018

^b^Data reported for 2014 and onward

**Table 3:** Rate of rape-related crimes per 100,000 women and girls reported in NCRB by states of India in 2018. SDI denotes Socio-demographic Index

India/states	Crime rate per 100,000 population in women and girls (95% confidence interval)					
	All rape-related crimes	Assault on women with intent to outrage her modesty	Rape	Insult to the modesty of women	Murder with gang rape	Attempt to commit rape
**India**	19.8 (19.7–19.9)	13.2 (13.1–13.3)	4.9 (4.9–5.0)	1.04 (1.01–1.06)	0.04 (0.04–0.05)	0.61 (0.59–0.63)
**Low SDI states**	20.7 (20.6–20.9)	13.4 (13.3–13.6)	6.1 (6.0–6.2)	0.38 (0.36–0.40)	0.06 (0.05–0.06)	0.74 (0.71–0.77)
Bihar	1.82 (1.71–1.93)	0.45 (0.39–0.50)	1.11 (1.03–1.20)	0.05 (0.03–0.07)	0.02 (0.01–0.03)	0.19 (0.15–0.22)
Madhya Pradesh	34.6 (34.0–35.1)	20.8 (20.4–21.2)	12.8 (12.5–13.2)	0.64 (0.56–0.71)	0.11 (0.08–0.14)	0.17 (0.13–0.21)
Jharkhand	14.9 (14.3–15.5)	7.4 (7.0–7.8)	5.9 (5.5–6.2)	0.20 (0.14–0.26)	0.04 (0.01–0.07)	1.35 (1.19–1.52)
Uttar Pradesh	15.1 (14.8–15.3)	11.0 (10.8–11.2)	3.5 (3.3–3.6)	0.01 (0.01–0.02)	0.04 (0.02–0.05)	0.58 (0.53–0.62)
Rajasthan	26.3 (25.8–26.8)	13.5 (13.1–13.8)	11.1 (10.8–11.5)	0.09 (0.06–0.12)	0.02 (0.00–0.03)	1.59 (1.47–1.72)
Chhattisgarh	26.9 (26.1–27.8)	12.0 (11.4–12.5)	13.5 (12.9–14.1)	1.19 (1.02–1.36)	0.06 (0.02–0.10)	0.17 (0.11–0.24)
Odisha	49.4 (48.5–50.3)	42.7 (41.8–43.5)	3.9 (3.7–4.2)	2.2 (2.0–2.4)	0.00 (0.00–0.01)	0.67 (0.56–0.77)
Assam	37.3 (36.4–38.2)	23.6 (22.9–24.4)	9.3 (8.9–9.8)	0.97 (0.82–1.11)	0.37 (0.28–0.46)	3.0 (2.7–3.2)
**Middle SDI states**	18.0 (17.8–18.2)	12.4 (12.3–12.6)	3.0 (2.9–3.0)	1.84 (1.78–1.90)	0.04 (0.03–0.04)	0.77 (0.73–0.81)
Andhra Pradesh	27.3 (26.6–27.9)	16.3 (15.9–16.8)	3.6 (3.3–3.8)	6.6 (6.3–6.9)	0.01 (0.00–0.03)	0.70 (0.60–0.80)
West Bengal	11.8 (11.5–12.1)	6.9 (6.7–7.2)	2.2 (2.0–2.3)	0.80 (0.72–0.88)	0.01 (0.00–0.02)	1.92 (1.80–2.05)
Tripura	15.1 (13.4–16.8)	8.3 (7.0–9.5)	4.9 (3.9–5.8)	0.30 (0.06–0.54)	0	1.65 (1.09–2.22)
Arunachal Pradesh	23.1 (19.9–26.4)	12.4 (10.0–14.7)	8.0 (6.1–10.0)	1.44 (0.62–2.25)	0	1.32 (0.54–2.10)
Meghalaya	13.3 (11.5–15.0)	5.6 (4.5–6.8)	5.2 (4.1–6.2)	0.95 (0.48–1.41)	0.24 (0.00–0.47)	1.30 (0.76–1.85)
Karnataka	17.4 (17.0–17.9)	15.5 (15.1–15.9)	1.46 (1.34–1.59)	0.41 (0.34–0.48)	0.05 (0.02–0.07)	0.03 (0.01–0.05)
Telangana	31.1 (30.3–31.9)	23.3 (22.6–24.0)	3.1 (2.8–3.3)	4.5 (4.2–4.8)	0.03 (0.01–0.06)	0.19 (0.13–0.26)
Gujarat	5.4 (5.1–5.6)	3.6 (3.4–3.8)	1.66 (1.52–1.80)	0.04 (0.02–0.06)	0.02 (0.00–0.03)	0.01 (0.00–0.02)
Manipur	7.1 (5.8–8.3)	3.3 (2.4–4.1)	3.0 (2.2–3.8)	0.46 (0.14–0.79)	0	0.35 (0.07–0.63)
Jammu & Kashmir	29.8 (28.5–31.1)	24.2 (23.0–25.4)	4.9 (4.3–5.4)	0.38 (0.23–0.53)	0	0.35 (0.21–0.49)
Haryana	31.8 (30.9–32.7)	19.5 (18.7–20.2)	9.5 (8.9–10.0)	1.40 (1.20–1.60)	0.19 (0.12–0.26)	1.28 (1.09–1.47)
**High SDI states**	20.1 (19.9–20.3)	13.6 (13.5–13.8)	4.9 (4.8–5.0)	1.45 (1.39–1.50)	0.03 (0.02–0.03)	0.14 (0.12–0.15)
Uttarakhand	20.0 (18.9–21.2)	9.6 (8.8–10.5)	9.9 (9.1–10.7)	0.14 (0.04–0.24)	0.04 (−0.01–0.08)	0.33 (0.18–0.48)
Tamil Nadu	2.9 (2.8–3.1)	2.0 (1.9–2.2)	0.82 (0.74–0.91)	0.03 (0.02–0.05)	0.01 (0.00–0.02)	0.03 (0.01–0.05)
Mizoram	19.0 (15.6–22.4)	10.7 (8.1–13.3)	8.0 (5.8–10.2)	0.16 (−0.15–0.47)	0.16 (− 0.15–0.47)	0.00 (0.00–0.00)
Maharashtra	23.3 (22.9–23.7)	18.0 (17.6–18.3)	3.6 (3.4–3.7)	1.78 (1.67–1.89)	0.03 (0.01–0.04)	0.01 (0.00–0.01)
Punjab	12.8 (12.3–13.4)	6.5 (6.1–6.9)	5.7 (5.3–6.0)	0.13 (0.07–0.19)	0.03 (0.00–0.05)	0.54 (0.42–0.66)
Sikkim	14.2 (10.0–18.3)	7.2 (4.3–10.2)	5.0 (2.6–7.5)	1.57 (0.19–2.95)	0	0.31 (−0.30–0.93)
Nagaland	2.6 (1.6–3.6)	1.05 (0.40–1.70)	1.05 (0.40–1.70)	0.52 (0.06–0.98)	0	0.00 (0.00–0.00)
Himachal Pradesh	25.4 (23.8–27.1)	13.7 (12.5–14.9)	9.2 (8.2–10.2)	2.1 (1.6–2.5)	0.19 (0.05–0.33)	0.29 (0.12–0.47)
Union territories other than Delhi	20.9 (18.8–23.0)	11.6 (10.0–13.2)	7.2 (5.9–8.4)	1.12 (0.63–1.62)	0	1.01 (0.54–1.48)
Kerala	38.0 (37.1–38.9)	24.7 (23.9–25.4)	10.6 (10.1–11.0)	2.5 (2.3–2.7)	0.01 (−0.01–0.02)	0.31 (0.23–0.40)
Delhi	49.3 (47.8–50.7)	29.7 (28.6–30.8)	13.3 (12.6–14.1)	6.1 (5.6–6.6)	0.03 (0.00–0.07)	0.12 (0.05–0.19)
Goa	28.0 (24.2–31.7)	16.4 (13.5–19.3)	8.0 (6.0–10.0)	3.4 (2.1–4.7)	0.13 (−0.13–0.39)	0.00 (0.00–0.00)

Almost all of the increase in the all rape-related crime rate seen between 2001 and 2018 (70.7%) was after 2012 (56.4% increase between 2013 and 2018; Supplementary Table 3). However, the state-level data highlight that not all states registered this increase, but we also didn’t see any definite pattern across some states for a meaningful understanding of the trend over time. The states of Assam (69.5%), Odisha (89.4%), Rajasthan (111·6%), Jharkhand (131%), and Uttar Pradesh (197.9%) registered a notable increase post 2012 in the low SDI states (Supplementary Fig. 1); Haryana (148·1%) and Karnataka (50%) in the middle SDI states (Supplementary Fig. 2); and Delhi (148·1%), Goa (73.9%), Kerala (31.5%) and Maharashtra (87.9%) in the high SDI states (Supplementary Fig. 3). Interestingly, many states had nearly stagnant rate over time. These included Bihar, Madhya Pradesh, Gujarat, Manipur, and Nagaland. The trend in Delhi stood out with the increase in rate after 2012 almost double that of other states, which then plateaued at 49.3 (Supplementary Fig. 3).

### Assault on women with intent to outrage her modesty

**Fig. 2:**
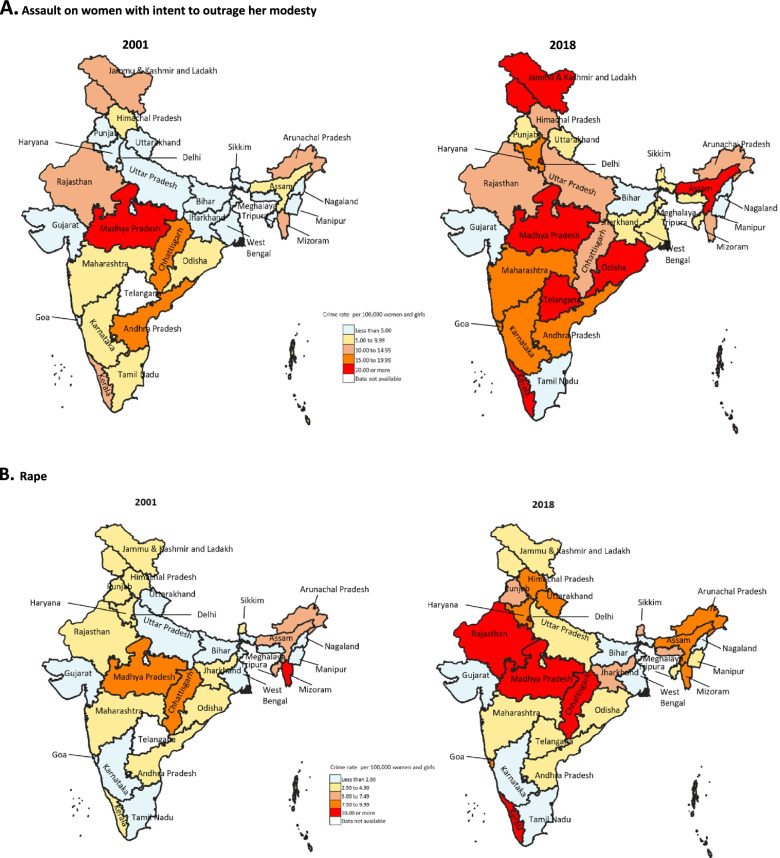
**A**. Crime rate for assault on women with intent to outrage her modesty per 100,000 women and girls for 2001 and 2018, by state. **B**. Crime rate for rape per 100,000 women and girls for 2001 and 2018, by state

Assault on women with intent to outrage her modesty accounted for 948,395 (59·4%) of all the rape-related crime cases reported between 2001 and 2018 in India. The rate of this crime was 6.6 (95% CI 6.5–6.7) in 2001 and 13.2 (95% CI 13.1–13.3) in 2018 per 100,000 women and girls (Table 2 and Fig. 1), a significant increase of 99.7%. In 2018, it ranged from 0·4 (95% CI 0·4–0.5) in Bihar to 42.7 (95% CI 41.8–3.5) in Odisha (Table 3), with nearly similar rates in the low-, middle-, and high-SDI states (Table 2). The state of Goa documented the highest percent increase at 559·4% (95% CI 434·4–684·3) over these years, 5.6 times higher than the national average increase (Fig. 2A and Supplementary Table 3). Nine states documented more than 200% increase in this rate between 2001 to 2018 and the highest decline of 67·2% (95% CI − 76.3 to − 58·0) was documented for Bihar (Supplementary Table 3). The significant increase in the all rape-related crimes rate seen after 2012 largely resulted from increase in the assault on women with intent to outrage her modesty (Fig. 1 and Supplementary Table 3).

The mean number of persons arrested for this crime remained stagnant over the years, and was 1·2 and 1·1 in 2001 and 2018 in India, respectively (Supplementary Fig. 4). A dip was seen in 2016 in the data which was likely due to data non-availability for that year. The findings were similar for the states by SDI groups from 2001 to 2015, and ranged from 0·6 in Manipur to 1·9 in Jammu & Kashmir in 2015 (data not shown).

### Rape

A total of 449,092 (28·1%) rape cases were reported in India between 2001 and 2018. The rate of rape crime was 3.1 (95% CI 3.1–3.2) in 2001 and 4.9 (95% CI 4·9–5·0) per 100,000 women and girls in 2018 (Table 2 and Fig. 1), exhibiting a significant increase of 58.7% (95% CI 56·4–61·0). This rate in 2018 ranged from 0.8 (95% CI 0.7–0.9) in Tamil Nadu to 13.5 (95% CI 12.9–14.1) in Chhattisgarh (Table 3), with it being higher in the low SDI states (6.1; 95% CI 6.0–6.2) as compared with the middle and high SDI states (Table 2). The highest percent increase from 2001 to 2018 was 484·2% (95% CI 430·8–537·7) in Uttarakhand, eight times the national average increase, and Kerala and Goa which documented more than a 200% percent increase (Fig. 2B and Supplementary Table 3). The highest decline was documented for Bihar (48·4%; 95% CI − 56·0 to − 40·7; Supplementary Table 3).

**Fig. 3:**
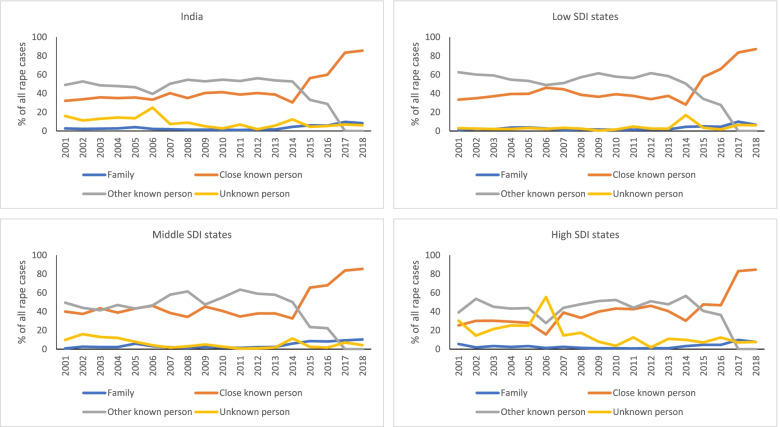
Yearly trends in the offender type for rape cases reported in NCRB 2001–2018. SDI denotes Socio-demographic Index

The mean number of persons arrested for rape in India also remained stagnant over the years, with it being 1·2 in 2018 and 1·3 in 2001 and a dip in 2016 (Supplementary Fig. 4), and ranged from 0·7 in Manipur to 1·9 in Karnataka in 2015 (data not shown). Data on the type of offender was available for all rape cases reported between 2001 and 2018. Family and unknown offender accounted for the least cases over this period, and a close known person or a known person accounted for the majority (Fig. 3). The data pattern highlighted possible issues in categorisation of the type of offender across the administrative splits. A relatively higher proportion of cases were reported as “other known person” as compared with “close known person” until 2014, however, after that a substantial increase was seen in cases reported as “close known person” with a steep decline in the reporting of “other known person”. The pattern of type of offender for rape was similar across the SDI groups, with only a higher reporting of “unknown person” in the high SDI states group until 2007 (Fig. 3).

### Insult to the modesty of women

A total of 176,811 (11·1%) cases were registered under ‘insult to the modesty of women’ in India between 2001 and 2018. The rate of this crime was 1.90 (95% CI 1.89–1.93) in 2001 and 1.04 (95% CI 1·01–1·06) in 2018 per 100,000 women and girls (Table 2 and Fig. 1).

### Status of the legal cases

Of the 592,611 cases undergoing a trial in India in 2018, the trial was listed as completed only in 56,821 (9·6%) cases at the end of 2018. Among the 56,821 completed trials, the offender(s) were acquitted or discharged in 41,950 (73%) cases.

## Discussion

The NCRB data from 2001 to 2018 documented an increase of 70.6% in the reported all rape-related crime rate, with most of this increase post 2012. The all rape-related crime rate was 19.8 per 100,000 women and girls in 2018 in India, with similar rates across the three SDI categories of the states. Assault with the intent to outrage modesty of the woman and rape formed majority of the reported crime categories, and close known and other known people were the most reported offenders with some variation seen over time. The legal process of trial completion and conviction leaves much to be desired. We discuss these findings to draw attention to the areas of the greatest need in data for prevention of such crimes in India to reach the SDG target of eliminating violence against women and girls.

The NCRB data are based on the *First Information Report* (FIR) filed with the police for a crime, and these data are known to under-report a variety of crimes including sexual violence [30, 31]. It is estimated that only 1 % of victims report sexual violence to the authorities [4, 32], due to various reasons such as the false beliefs around sexual violence that place responsibility on the victim, and stigma around rape and female sexuality in India [33]. Reporting is also discouraged as the women and girls are afraid of being ostracised or disbelieved [34, 35]. The implication of Principle Offence Rule followed by the NCRB in reporting, which is also followed in many developed countries, is also one of the reasons for under-reporting [22, 36]. According to this rule, only the crime that attracts maximum punishment is considered for reporting in NCRB irrespective of the number of offences registered in a single FIR case [36, 37]. For example, an incident involving abduction, wrongful confinement, rape, and murder will be reported in NCRB as murder as it warrants the maximum punishment which is death sentence. Following this rule, the Nirbhaya case, and the recent gang-rape cases were recorded as murder and not as rape [9, 11, 12]. Therefore, it is very likely that there are more rape-related cases in the police records which are hidden in the NCRB reports due to the reporting procedure followed.

Comparison of the all rape-related crime rate of 0.02% for all age women in NCRB with 5.2% reported in the National Family Health Survey-4 (NHFS-4) for 15–49 years women further highlights the challenges in fully understanding the burden of sexual violence in India [38]. In addition to the former being from the police records for women of all ages and the latter from population-based assessment predominately of ever married women in 15–49 years age group, one of the major reasons for such extreme differences in these two data sources is the type of sexual crime that is captured. Among the ever married women aged 15–49 years women in NFHS-4 who had reported sexual violence, 92% of them had reported their current or former husband as the offender for sexual violence [38]. However, such cases are rarely reported to the police since marital rape is not considered a crime in India [39].

The steep increase to the otherwise stagnant rate in the NCRB data seen after 2012 is widely attributed to increased reporting rather than increased incidence of these crimes. The rise in public discussion of crimes against women and the legal changes made following the *Nirbhaya* case of December 2012 are known to have encouraged willingness to report amongst victims [4, 40]. Furthermore, some increase in the number of cases reported could also be due to the 2013 Criminal Law Amendment which explicitly recognised more acts as sexual violence in addition to peno-vaginal penetration including anal sex, oral sex, use of objects including finger [8]. There remains a possibility of increase in the rape-related crime over the years in addition to the increased reporting, however, NCRB data do not allow for such an understanding. Interestingly, a decline in the prevalence of sexual violence was reported between the two rounds of NFHS, 2005–06 and 2014–15, among women aged 15–49 years [38].

Slightly but significantly higher rate of sexual violence in low SDI states can be understood through a combination of the general strain theory, wherein crime increases when the population fails to meet its aspirations [41], and prevalent gender norms that award lower status to women and girls [42, 43], and normalise gender-based violence [32]. In the high SDI states, empowerment through higher educational attainment of girls might result in less conformity to patriarchal norms, thereby, straining the social norms and inciting men towards sexual violence to maintain the power structure [32]. However, the understanding of the context of these crimes is limited due to non-availability of the individual level data on education and social status of the victim and the offender in NCRB. Furthermore, understanding of the wide state-level variability in the crime rate across years in some states from 2001 to 2018, and that of the directionality of change post 2012 is also impeded by the lack of individual data. Previous reports indicate that the increase in reporting of sexual violence post the *Nirbhaya* case was seen in states that were closer to New Delhi [4]. This may explain the higher increases in the states of Delhi, Haryana, Rajasthan and Uttar Pradesh. However, particularly in Delhi, we see that this momentum did not sustain, and dropped from the rate of 105 per 100,000 women in 2015 to 49 in 2018. Prior research evidences that the increased reporting of the crime which was driven by more awareness and promise of faster and effective justice saw a steep fall after a few years due to lack of actual improvements and implementation of promises by the government [44].

Contradictory to the popular myth that rape is done by strangers [45], a known person was reported as an offender in majority of the reported rape cases. However, this finding is in contrast to the previous suggestion of a direct association of relational distance with reporting, whereby known persons are least likely to be reported [46]. It is also possible that the cases where the offender is not known are reported less to the police as there is no person who can be named in the police report. Such a pattern of under-reporting is documented in the “hit and run” road traffic crash cases in the NCRB data wherein the details of the other person or vehicle are not readily available [30]. Finally, the expansion of the close known person category in 2014 by the NCRB might have also increased representation of such cases [47]. However, better understanding of the offenders is needed as it has direct implications for prevention measures.

Despite the increased legal attention on sexual crimes in India post 2012, the legal system leaves much to be desired with only 10 % of the registered trials reported as completed. As an indicator of the scope of the problem of rape prosecution, the *Nirbhaya* case was the only conviction obtained among the 706 rape cases filed in Delhi in 2012 [48]. Significant delays are known in the Indian judicial system [49, 50], and these not only emotionally impacts the victim and their family, but also discourage reporting. It is difficult to quantify the length of trials or waiting, as such data are not readily available. This barrier to reporting might be further compounded by the low conviction rate as we found that 75% of the accused were acquitted. This might incentivise crime itself, as per the Rational Cost Theory which indicates increase in crime when the risk of punishment is low [51].

These findings should be viewed in light of some limitations of the NCRB data. As indicated earlier, though under-reporting is well known in the NCRB data, it is not possible for us to comment on the extent of underreporting of data or the pattern of underreporting by type of crime, year or state. A recent comparison of the NCRB data on suicide deaths with that from the GBD Study has highlighted wide state-level variations in underreporting in the former [31]. It has also been suggested that under-reporting at the population level could be because of the mandatory reporting of sexual violence cases in India, [23, 24, 52] which also in turn results in poor access to treatment and support services for the victims [53].

The NCRB data are a passive form of surveillance dependent on the availability and quality of data recorded by the police in the FIR at the local level [22]. Despite the availability of POSCO Act to register sexual offences against victims aged < 18 years, these cases continued to be registered under the other crime categories for women. There appears to be less clear distinction or an overlap in use of crime categories to register FIR, however, the reasons for these are beyond the scope of this paper. Age was not available for all the crime categories of interest, and the age category documentation in rape category showed overlap within the categories (for example, 10–14 years and 14–16 years), thereby, limiting the utilisation of these data for meaningful interpretation. Therefore, significant improvements in the FIR reporting of the rape-related crimes are urgently needed for these data to provide in-depth insights for developing effective prevention strategies against sexual violence against women and girls. The World Health Organization injury surveillance guidelines could be urgently considered for collecting systematic data on sexual violence in the police records [54]. The NCRB should also consider making available de-identified individual level data to allow for analysis that can highlight possible risk factors to prevent such crimes.

## Conclusions

India’s ability to respond to SDG target of eliminating all forms of violence against women and girls will remain severely restricted unless the data gaps in the administrative data are not urgently addressed. Understanding and removing barriers to reporting, improving quality of data recorded, and expanding scope of data collected is urgently needed for formulating well-informed public health intervention strategies to reduce sexual violence against women and girls in India.

## Supplementary Information

**Additional file 1: Supplementary Figure 1.** All rape-related crime rate per 100,000 women and girls in the states categorised as having low Socio-demographic Index, 2001–2018.

**Additional file 2: Supplementary Figure 2.** All rape-related crime rate per 100,000 women and girls in the states categorised as having middle Socio-demographic Index, 2001–2018.

**Additional file 3: Supplementary Figure 3.** All rape-related crime rate per 100,000 women and girls in the states categorised as having high Socio-demographic Index, 2001–2018.

**Additional file 4: Supplementary Figure 4.** Mean number of persons arrested for ‘assault on women with intent to outrage her modesty’ and ‘rape’ 2001–2018. SDI denotes Socio-demographic Index. The state wise data for mean number of persons was available from 2001 to 2015 only.

**Additional file 5: Supplementary Table 1.** Definitions of the crime categories as per the Indian Penal Code (IPC).

**Additional file 6: Supplementary Table 2.** Age data availability in National Crimes Records Bureau reports for women and girls by the types of rape-related crimes from 2001 to 2018.

**Additional file 7: Supplementary Table 3.** Percent change in the crime rates of all rape-related crimes, assault of women with intent to outrage her modesty, and rape per 100,000 women and girls between 2001 and 2018, 2012 and 2018, and 2001 and 2018 in India and its states categorised by Socio-demographic Index (SDI).

## Data Availability

The rape related data used in these analyses are available at NCRB website (https://ncrb.gov.in) and from G. Anil Kumar on request. The GBD population data used in these analyses are available at http://ghdx.healthdata.org/gbd%C2%AD2019.

## Abbreviations

IPV: Intimate Partner Violence
SDG: Sustainable Development Goal
NFHS: National Family Health Survey
NCRB: National Crime Record Bureau
SDI: Socio-demographic Index
GBD: Global Burden of Disease
FIR: First Information Report
