# Supplementary material for: Administrative data deficiencies plague understanding of the magnitude of rape-related crimes in Indian women and girls
Source: BMC Public Health. 2022 Apr 19;22:788. doi: 10.1186/s12889-022-13182-0 (PMC9020006; doi:10.1186/s12889-022-13182-0)
Supplement: Supplementary file 1 — Additional file 1: Supplementary Figure 1. All rape-related crime rate per 100,000 women and girls in the states categorised as having low Socio-demographic Index, 2001–2018. [file 12889_2022_13182_MOESM1_ESM.docx]

**Supplementary Figure 1 –** All rape-related crime rate per 100,000 women and girls in the states categorised as having low Socio-demographic Index, 2001-2018.
