# Supplementary material for: Administrative data deficiencies plague understanding of the magnitude of rape-related crimes in Indian women and girls
Source: BMC Public Health. 2022 Apr 19;22:788. doi: 10.1186/s12889-022-13182-0 (PMC9020006; doi:10.1186/s12889-022-13182-0)
Supplement: Supplementary file 4 — Additional file 4: Supplementary Figure 4. Mean number of persons arrested for ‘assault on women with intent to outrage her modesty’ and ‘rape’ 2001–2018. SDI denotes Socio-demographic Index. The state wise data for mean number of persons was available from 2001 to 2015 only. [file 12889_2022_13182_MOESM4_ESM.docx]

**Supplementary Figure 4** – Mean number of persons arrested for ‘assault on women with intent to outrage her modesty’ and ‘rape’ 2001-2018. SDI denotes Socio-demographic Index. The state wise data for mean number of persons was available from 2001-2015 only.
