# Supplementary material for: Administrative data deficiencies plague understanding of the magnitude of rape-related crimes in Indian women and girls
Source: BMC Public Health. 2022 Apr 19;22:788. doi: 10.1186/s12889-022-13182-0 (PMC9020006; doi:10.1186/s12889-022-13182-0)
Supplement: Supplementary file 5 — Additional file 5: Supplementary Table 1. Definitions of the crime categories as per the Indian Penal Code (IPC). [file 12889_2022_13182_MOESM5_ESM.docx]

Supplementary Table 1. Definitions of the crime categories as per the Indian Penal Code (IPC).

| **Crime category** | **IPC section** | **Definition** |
| --- | --- | --- |
| Assault on women with intent to outrage her modesty | 354 | Whoever assaults or uses criminal force to any woman, intending to outrage or knowing it to be likely that he will thereby outrage her modesty, shall be punished with imprisonment of either description for a term which shall not be less than one year but which may extend to five years, and shall also be liable to fine. |
| Rape | 375 | A man is said to commit “rape” who, except in the case hereinafter excepted, has sexual intercourse with a woman under circumstances falling under any of the six following descriptions:  (First) — Against her will.  (Secondly) —Without her consent.  (Thirdly) — With her consent, when her consent has been obtained by putting her or any person in whom she is interested in fear of death or of hurt.  (Fourthly) —With her consent, when the man knows that he is not her husband, and that her consent is given because she believes that he is another man to whom she is or believes herself to be law­fully married.  (Fifthly) — With her consent, when, at the time of giving such consent, by reason of unsoundness of mind or intoxication or the administration by him personally or through another of any stupe­fying or unwholesome substance, she is unable to understand the nature and consequences of that to which she gives consent.  (Sixthly) — With or without her consent, when she is under eighteen years of age.  Seventhly.—When she is unable to communicate consent.  Explanation 1.—For the purposes of this section, “vagina” shall also include labia majora.  Explanation 2.—Consent means an unequivocal voluntary agreement when the woman by words, gestures or any form of verbal or non-verbal communication, communicates willingness to participate in  the specific sexual act:  Provided that a woman who does not physically resist to the act of penetration shall not by the reason only of that fact, be regarded as consenting to the sexual activity.  Exception 1.—A medical procedure or intervention shall not constitute rape.  Exception 2.—Sexual intercourse or sexual acts by a man with his own wife, the wife not being under fifteen years of age, is not rape |
| Insult to the modesty of women | 509 | Whoever, intending to insult the modesty of any woman, utters any word, makes any sound or gesture, or exhibits any object, intending that such word or sound shall be heard, or that such gesture or object shall be seen, by such woman, or intrudes upon the privacy of such woman, shall be punished with simple imprisonment for a term which may extend to three years, and also with fine. |
| Murder with rape/gang rape | 302  375  376(2)(g) | Whoever commits murder shall be punished with death, or imprisonment for life, and shall also be liable to fine.  As indicated above.  Where a woman is raped by one or more persons constituting a group or acting in furtherance of a common intention, each of those persons shall be deemed to have committed the offence of rape, shall be punished with rigorous imprisonment for a term which shall not be less than twenty years but which may extend to life which shall mean imprisonment for the remainder of that person's natural life, and with fine:  Provided that such fine shall be just and reasonable to meet the medical expenses and rehabilitation of the victim:  Provided further that any fine imposed under this section shall be paid to the victim. |
| Attempt to rape | 376  511  354 | Deals with punishments for rape.  Whoever attempts to commit an offence punishable by this Code with  [imprisonment for life] or imprisonment, or to cause such an offence to be committed, and in such attempt does any act towards the commission of the offence, shall, where no express provision is made by this Code for the punishment of such attempt, be punished with [imprisonment of any description provided for the offence, for a term which may extend to one-half of the imprisonment for life or, as the case may be, one- half of the longest term of imprisonment provided for that offence], or with such fine as is provided for the offence, or with both.  As indicated above. |
