# Supplementary material for: Administrative data deficiencies plague understanding of the magnitude of rape-related crimes in Indian women and girls
Source: BMC Public Health. 2022 Apr 19;22:788. doi: 10.1186/s12889-022-13182-0 (PMC9020006; doi:10.1186/s12889-022-13182-0)
Supplement: Supplementary file 6 — Additional file 6: Supplementary Table 2. Age data availability in National Crimes Records Bureau reports for women and girls by the types of rape-related crimes from 2001 to 2018. [file 12889_2022_13182_MOESM6_ESM.docx]

**Supplementary Table 2 –** Age data availability in National Crimes Records Bureau reports for women and girls by the types of rape-related crimes from 2001-2018.

| **Year** | **Age data availability (in years)** | | | | |
| --- | --- | --- | --- | --- | --- |
|  | **Assault on women with intent to outrage her modesty** | **Rape** | **Insult to the modesty of women** | **Murder with gang rape** | **Attempt to commit rape** |
| 2001 | Not available | Upto 10, 10-14, 14-18,18-30, 30-50, above 50 | Not available | Not available | Not available |
| 2002 | Not available | Upto 10, 10-14, 14-18,18-30, 30-50, above 50 | Not available | Not available | Not available |
| 2003 | Not available | Upto 10, 10-14, 14-18,18-30, 30-50, above 50 | Not available | Not available | Not available |
| 2004 | Not available | Upto 10, 10-14, 14-18,18-30, 30-50, above 50 | Not available | Not available | Not available |
| 2005 | Not available | Upto 10, 10-14, 14-18,18-30, 30-50, above 50 | Not available | Not available | Not available |
| 2006 | Not available | Upto 10, 10-14, 14-18,18-30, 30-50, above 50 | Not available | Not available | Not available |
| 2007 | Not available | Upto 10, 10-14, 14-18,18-30, 30-50, above 50 | Not available | Not available | Not available |
| 2008 | Not available | Upto 10, 10-14, 14-18,18-30, 30-50, above 50 | Not available | Not available | Not available |
| 2009 | Not available | Upto 10, 10-14, 14-18,18-30, 30-50, above 50 | Not available | Not available | Not available |
| 2010 | Not available | Upto 10, 10-14, 14-18,18-30, 30-50, above 50 | Not available | Not available | Not available |
| 2011 | Not available | Upto 10, 10-14, 14-18,18-30, 30-50, above 50 | Not available | Not available | Not available |
| 2012 | Not available | Upto 10, 10-14, 14-18,18-30, 30-50, above 50 | Not available | Not available | Not available |
| 2013 | Not available | Upto 10, 10-14, 14-18,18-30, 30-50, above 50 | Not available | Not available | Not available |
| 2014 | Not available | Below 6, 6-11, 12-15, 16-17, 18-29, 30-44, 45-59, above 60 | Not available | Not available | Not available |
| 2015 | Not available | Below 6, 6-11, 12-15, 16-17, 18-29, 30-44, 45-59, above 60 | Not available | Not available | Not available |
| 2016 | Not available | Below 6, 6-11, 12-15, 16-17, 18-29, 30-44, 45-59, above 60 | Not available | Not available | Not available |
| 2017 | <18, >18 | Below 6, 6-11, 12-15, 16-17, 18-29, 30-44, 45-59, above 60 | <18, >18 | Not available | <18, >18 |
| 2018 | <18, >18 | Below 6, 6-11, 12-15, 16-17, 18-29, 30-44, 45-59, above 60 | <18, >18 | Not available | <18, >18 |
