# Supplementary material for: Administrative data deficiencies plague understanding of the magnitude of rape-related crimes in Indian women and girls
Source: BMC Public Health. 2022 Apr 19;22:788. doi: 10.1186/s12889-022-13182-0 (PMC9020006; doi:10.1186/s12889-022-13182-0)
Supplement: Supplementary file 7 — Additional file 7: Supplementary Table 3. Percent change in the crime rates of all rape-related crimes, assault of women with intent to outrage her modesty, and rape per 100,000 women and girls between 2001 and 2018, 2012 and 2018, and 2001 and 2018 in India and its states categorised by Socio-demographic Index (SDI). [file 12889_2022_13182_MOESM7_ESM.docx]

**Supplementary Table 3 –** Percent change in the crime rates of all rape-related crimes, assault of women with intent to outrage her modesty, and rape per 100,000 women and girls between 2001 and 2018, 2012 and 2018, and 2001 and 2018 in India and its states categorised by Socio-demographic Index (SDI).

| **States of India** | **All rape related crimes** | | | | | | **Assault of women with intent to outrage her modesty** | | | | | | **Rape** | | | | | |
| --- | --- | --- | --- | --- | --- | --- | --- | --- | --- | --- | --- | --- | --- | --- | --- | --- | --- | --- |
|  | **2001** | **2012** | **2018** | **% change 2001-2012** | **% change 2012-2018** | **% change 2001-2018** | **2001** | **2012** | **2018** | **% change 2001-2012** | **% change 2012-2018** | **% change 2001-2018** | **2001** | **2012** | **2018** | **% change 2001-2012** | **% change 2012-2018** | **% change 2001-2018** |
| **India** | 11.6 | 12.7 | 19.8 | 9.2 | 56.4 | 70.8 | 6.6 | 7.2 | 13.2 | 9.5 | 82.4 | 99.7 | 3.1 | 4.0 | 4.9 | 27.7 | 24.3 | 58.7 |
| **Low SDI states** | 13.4 | 11.7 | 20.7 | -12.6 | 77.4 | 55 | 7.5 | 6.8 | 13.4 | -10.1 | 98.5 | 78.5 | 4.1 | 4.5 | 6.1 | 7.7 | 36.7 | 47.3 |
| Bihar | 3.6 | 2.0 | 1.8 | -43.4 | -10.1 | -49.1 | 1.4 | 0.2 | 0.4 | -83.8 | 103.3 | -67.2 | 2.2 | 1.7 | 1.1 | -19.7 | -35.7 | -48.4 |
| Madhya Pradesh | 35.6 | 28.7 | 34.6 | -19.5 | 20.5 | -2.9 | 23.6 | 17.6 | 20.8 | -25.4 | 18.2 | -11.8 | 9.5 | 9.0 | 12.8 | -4.9 | 42.0 | 35 |
| Jharkhand | 6.4 | 6.5 | 14.9 | 0.9 | 131.0 | 133.1 | 2.2 | 1.7 | 7.4 | -24.2 | 348.7 | 240.1 | 4.2 | 4.7 | 5.9 | 13.5 | 24.1 | 40.9 |
| Uttar Pradesh | 9.0 | 5.1 | 15.1 | -43.8 | 197.9 | 67.3 | 3.5 | 3.1 | 11.0 | -9.8 | 249.0 | 214.7 | 2.4 | 1.9 | 3.5 | -20.1 | 81.4 | 45 |
| Rajasthan | 14.2 | 12.4 | 26.3 | -12.3 | 111.6 | 85.6 | 10.2 | 6.6 | 13.5 | -35.4 | 103.7 | 31.6 | 3.7 | 5.8 | 11.1 | 54.4 | 93.1 | 198.2 |
| Chattisgarh | 26.6 | 20.3 | 26.9 | -23.8 | 32.9 | 1.2 | 16.3 | 11.6 | 12.0 | -28.7 | 3.3 | -26.3 | 8.9 | 7.5 | 13.5 | -15.3 | 80.4 | 52.7 |
| Odisha | 15.3 | 26.8 | 49.4 | 75.2 | 84.4 | 223 | 8.7 | 18.9 | 42.7 | 116.2 | 126.2 | 389.1 | 4.2 | 6.6 | 3.9 | 57.7 | -40.2 | -5.7 |
| Assam | 12.6 | 22.0 | 37.3 | 74.3 | 69.5 | 195.3 | 6.4 | 11.4 | 23.6 | 77.0 | 108.0 | 268.1 | 6.2 | 10.6 | 9.3 | 71.8 | -12.1 | 51 |
| **Middle SDI states** | 7.5 | 14.2 | 14.5 | 50.0 | 27.0 | 92.4 | 4.3 | 7.9 | 10.0 | 46.3 | 56.5 | 131.3 | 1.5 | 3.3 | 2.4 | 73.0 | -10.9 | 55.7 |
| Andhra Pradesh | 28.8 | 37.8 | 27.3 | 31.3 | -27.9 | -5.4 | 15.3 | 18.4 | 16.3 | 20.8 | -11.4 | 7 | 3.8 | 5.1 | 3.6 | 36.9 | -30.5 | -4.9 |
| West Bengal | 4.3 | 12.7 | 11.8 | 196.0 | -6.7 | 176.1 | 2.4 | 7.1 | 6.9 | 198.6 | -3.0 | 189.7 | 1.8 | 4.4 | 2.2 | 145.8 | -50.1 | 22.6 |
| Tripura | 10.0 | 29.0 | 15.1 | 189.9 | -48.0 | 50.7 | 3.6 | 16.6 | 8.3 | 356.6 | -50.1 | 127.9 | 6.4 | 12.1 | 4.9 | 89.3 | -59.8 | -23.8 |
| Arunachal Pradesh | 21.7 | 15.8 | 23.1 | -26.9 | 46.1 | 6.8 | 14.8 | 9.2 | 12.4 | -37.8 | 33.8 | -16.7 | 6.3 | 6.3 | 8.0 | 1.0 | 26.8 | 28 |
| Meghalaya | 4.3 | 13.6 | 13.3 | 213.5 | -2.1 | 206.9 | 2.1 | 2.8 | 5.6 | 32.9 | 99.8 | 165.5 | 2.2 | 10.7 | 5.2 | 387.2 | -52.0 | 133.8 |
| Karnataka | 7.6 | 11.6 | 17.4 | 53.6 | 50.0 | 130.4 | 6.2 | 9.4 | 15.5 | 51.5 | 65.4 | 150.6 | 1.1 | 2.0 | 1.5 | 79.5 | -25.0 | 34.6 |
| Telangana | - | NA | 31.1 | NA | NA | NA | - | NA | 23.3 | NA | NA | NA | - | NA | 3.1 | NA | NA | NA |
| Gujarat | 4.5 | 4.2 | 5.4 | -6.5 | 26.6 | 18.3 | 3.0 | 2.4 | 3.6 | -19.0 | 50.9 | 22.2 | 1.1 | 1.5 | 1.7 | 36.0 | 8.8 | 47.9 |

| Manipur | 3.6 | 7.4 | 7.1 | 105.8 | -4.7 | 96.2 | 1.9 | 3.2 | 3.3 | 74.3 | 0.9 | 75.8 | 1.8 | 4.1 | 3.0 | 135.3 | -27.1 | 71.4 |
| --- | --- | --- | --- | --- | --- | --- | --- | --- | --- | --- | --- | --- | --- | --- | --- | --- | --- | --- |
| Jammu and Kashmir1 | 21.7 | 32.1 | 29.8 | 48.0 | -7.3 | 37.2 | 12.5 | 21.5 | 24.2 | 72.1 | 12.4 | 93.4 | 3.4 | 4.9 | 4.9 | 45.2 | -1.7 | 42.6 |
| Haryana | 12.5 | 12.8 | 31.8 | 2.4 | 148.1 | 154 | 4.7 | 4.1 | 19.5 | -11.8 | 370.9 | 315.6 | 3.9 | 5.3 | 9.5 | 34.9 | 79.6 | 142.2 |
| **High SDI states** | 10.9 | 12.8 | 20.1 | 18.3 | 56.6 | 85.4 | 6.3 | 7.3 | 13.6 | 16.5 | 86.2 | 116.9 | 2.6 | 3.8 | 4.9 | 45.4 | 29.5 | 88.3 |
| Uttarakhand | 6.0 | 6.9 | 20.0 | 15.7 | 190.5 | 236 | 2.4 | 2.7 | 9.6 | 13.2 | 262.3 | 310 | 1.7 | 2.8 | 9.9 | 67.7 | 248.4 | 484.2 |
| Tamil Nadu | 9.9 | 6.9 | 2.9 | -30.6 | -57.3 | -70.3 | 5.5 | 3.9 | 2.0 | -28.2 | -48.3 | -62.9 | 1.3 | 1.9 | 0.8 | 48.5 | -57.4 | -36.7 |
| Mizoram | 23.6 | 33.4 | 19.0 | 41.7 | -43.1 | -19.4 | 11.8 | 15.1 | 10.7 | 28.1 | -29.1 | -9.2 | 11.8 | 18.3 | 8.0 | 55.2 | -56.3 | -32.2 |
| Maharashtra | 10.9 | 12.4 | 23.3 | 13.7 | 87.9 | 113.6 | 5.9 | 6.9 | 18.0 | 17.6 | 159.9 | 205.6 | 2.7 | 3.2 | 3.6 | 19.1 | 10.0 | 31 |
| Punjab | 6.0 | 7.6 | 12.8 | 25.5 | 69.5 | 112.7 | 3.1 | 2.5 | 6.5 | -21.7 | 165.1 | 107.5 | 2.5 | 4.9 | 5.7 | 95.4 | 15.2 | 125.1 |
| Sikkim | 8.5 | 17.3 | 14.2 | 104.9 | -18.2 | 67.5 | - | 6.2 | 7.2 | NA | 16.6 | NA | 3.1 | 11.1 | 5.0 | 261.4 | -54.7 | 63.8 |
| Nagaland | 2.3 | 3.8 | 2.6 | 60.6 | -30.3 | 12 | 0.6 | 1.6 | 1.0 | 166.3 | -35.5 | 71.8 | 1.7 | 2.1 | 1.0 | 23.4 | -50.8 | -39.4 |
| Himachal Pradesh | 14.4 | 14.1 | 25.4 | -1.9 | 80.7 | 77.2 | 9.9 | 7.0 | 13.7 | -29.3 | 94.9 | 37.9 | 4.0 | 5.1 | 9.2 | 29.4 | 78.6 | 131.1 |
| UTs other than Delhi | 12.6 | 10.1 | 20.9 | -20.1 | 107.5 | 65.9 | 6.5 | 4.5 | 11.6 | -30.8 | 156.2 | 77.2 | 2.8 | 3.7 | 7.2 | 32.4 | 96.4 | 160 |
| Kerala | 15.1 | 28.9 | 38.0 | 91.3 | 31.5 | 151.4 | 11.4 | 20.6 | 24.7 | 81.1 | 19.8 | 117 | 3.3 | 5.6 | 10.6 | 70.7 | 88.0 | 220.9 |
| Delhi | 15.2 | 19.9 | 49.3 | 30.6 | 148.1 | 224.1 | 7.8 | 8.8 | 29.7 | 12.2 | 237.6 | 278.8 | 6.0 | 8.5 | 13.3 | 43.5 | 56.2 | 124.2 |
| Goa | 5.3 | 16.1 | 28.0 | 205.0 | 73.9 | 430.6 | 2.5 | 6.6 | 16.4 | 163.8 | 150.0 | 559.4 | 1.8 | 7.4 | 8.0 | 319.4 | 8.7 | 355.8 |
